# Generating real‐world evidence in Alzheimer's disease: Considerations for establishing a core dataset

**DOI:** 10.1002/alz.13785

**Published:** 2024-05-06

**Authors:** James E. Galvin, Jeffrey L. Cummings, Mihaela Levitchi Benea, Carl de Moor, Ricardo F. Allegri, Alireza Atri, Howard Chertkow, Claire Paquet, Verna R. Porter, Craig W. Ritchie, Sietske A. M. Sikkes, Michael R. Smith, Christina Marsica Grassi, Ivana Rubino

**Affiliations:** ^1^ Comprehensive Center for Brain Health Department of Neurology University of Miami Miller School of Medicine Boca Raton Florida USA; ^2^ Chambers‐Grundy Center for Transformative Neuroscience Department of Brain Health University of Nevada Las Vegas (UNLV) Las Vegas Nevada USA; ^3^ Fulcrum Therapeutics Cambridge Massachusetts USA; ^4^ Biogen Cambridge Massachusetts USA; ^5^ Instituto de Investigaciones Neurológicas Fleni Buenos Aires Argentina; ^6^ Departamento de Neurociencias Universidad De La Costa (CUC), Barranquilla Atlántico Colombia; ^7^ Banner Sun Health Research Institute Sun City Arizona USA; ^8^ Center for Brain/Mind Medicine, Department of Neurology Brigham and Women's Hospital – Main Campus Boston Massachusetts USA; ^9^ Harvard Medical School Boston Massachusetts USA; ^10^ Rotman Research Institute, Baycrest Health Sciences Toronto Ontario Canada; ^11^ Université de Paris GHU AP‐HP Nord Lariboisière Hospital Paris France; ^12^ Pacific Brain Health Center, Pacific Neuroscience Institute Santa Monica California USA; ^13^ Saint John's Cancer Institute Santa Monica California USA; ^14^ Scottish Brain Sciences Edinburgh UK; ^15^ Alzheimer Center Amsterdam Amsterdam University Medical Center Amsterdam The Netherlands; ^16^ Department of Clinical, Neuro‐ and Developmental Psychology Vrije Universiteit (VU) Amsterdam Amsterdam The Netherlands

**Keywords:** Alzheimer's disease, core outcomes, data collection, population, real‐world evidence, study aims

## Abstract

Ongoing assessment of patients with Alzheimer's disease (AD) in postapproval studies is important for mapping disease progression and evaluating real‐world treatment effectiveness and safety. However, interpreting outcomes in the real world is challenging owing to variation in data collected across centers and specialties and greater heterogeneity of patients compared with trial participants. Here, we share considerations for observational postapproval studies designed to collect harmonized longitudinal data from individuals with mild cognitive impairment or mild dementia stage of disease who receive therapies targeting the underlying pathological processes of AD in routine practice. This paper considers key study design parameters, including proposed aims and objectives, study populations, approaches to data collection, and measures of cognition, functional abilities, neuropsychiatric status, quality of life, health economics, safety, and drug utilization. Postapproval studies that capture these considerations will be important to provide standardized data on AD treatment effectiveness and safety in real‐world settings.

## 1 BACKGROUND

Real‐world evidence (RWE) generation is becoming increasingly important to clinicians, regulatory agencies, and professional societies to inform patient access and understand the effectiveness, safety, and appropriate use of therapeutic agents.^1^, ^2^ RWE can also be used to support regulatory decision making, and the US Food and Drug Administration (FDA) has issued guidance on how RWE can help inform drug approval and support postapproval study requirements;^2^, ^3^, ^4^ real‐world data collection as presented here is one type of Phase IV study in a clinical development program. Registries represent another approach to RWE generation and the prescribing information (PI) for ADUHELM (aducanumab‐avwa) and for LEQEMBI (lecanemab‐irmb) include reference to the Alzheimer's Network for Treatment and Diagnostics (ALZ‐NET) as a voluntary patient registry that collects postmarketing information on treatments for Alzheimer's disease (AD).^5^, ^6^, ^7^ Registries, such as the ALZ‐NET registry, are data collection systems that capture a wide range of long‐term outcome data.^7^, ^8^ In contrast, both registry studies, such as the Centers for Medicare & Medicaid Services (CMS) National Patient Registry study, and Phase IV observational studies investigate a specific research question that determines the parameters for data collection.^8^, ^9^, ^10^ Both data from registries and postapproval studies can support regulatory decisions, but data from registries are generally more representative of the real‐world population than postapproval studies and are preferable for assessing outcomes in subpopulations not included in Phase III trials; postapproval studies are preferred for assessing the clinical benefit and treatment strategies for novel drugs.^11^ Since the US FDA accelerated approval of aducanumab and lecanemab (now traditionally approved) for the treatment of AD,^5^, ^6^, ^12^, ^13^ and with other anti‐amyloid beta (Aβ) monoclonal antibodies in clinical development, the focus on RWE generation in AD has intensified, specifically to understand the long‐term safety, effectiveness, and impact of anti‐Aβ monoclonal antibody treatment on patient‐related and healthcare resource utilization outcomes. In addition, Appropriate Use Recommendations for both aducanumab and lecanemab have been recently published to guide the introduction of these therapies into clinical practice. These recommendations highlight the need for careful patient selection, including the presence of amyloid pathology; the exclusion of patients at high risk of developing amyloid‐related imaging abnormalities (ARIA), such as those with cerebrovascular disease; recommendations for apolipoprotein E ε4 (*APOE* ε4) genotyping to assess ARIA risk; and monitoring of asymptomatic ARIA using serial magnetic resonance imaging.^14^, ^15^

Although randomized controlled trials (RCTs) are considered the gold‐standard study design for evidence‐based medicine, such studies have shortcomings in delivering generalizable information, as their methods can lack applicability in clinical practice.^1^, ^16^, ^17^ RCTs are typically conducted in academic research settings or specialized trial sites by highly trained clinicians, where extrinsic factors are controlled, regimen adherence monitored, and interventions provided free of charge.^17^, ^18^ Participant selection can be narrow and homogeneous, which limits the generalizability of results; specific patient populations are not routinely included in RCTs, such as those of advanced age and/or with comorbidities.^1^, ^17^, ^19^ African‐American, Hispanic, and Latino individuals have been historically underrepresented in AD clinical trials despite having a greater risk of developing AD than White individuals, and research has found that many commonly used exclusion criteria (eg, history of cardiovascular disease or diabetes) in AD clinical trials disproportionately affect African American, Hispanic, and Latino individuals.^20^, ^21^ In addition, treatment and follow‐up periods in RCTs are often short, potentially underestimating long‐term benefits and/or safety signals.^1^, ^18^

Real‐world studies aim to produce findings that translate research to routine practice, maximize generalizability, explore key treatment‐related factors driving clinical changes, and address considerations about the benefits, risks, and costs of an intervention.^17^ RWE can be used to confirm the effectiveness and tolerability of treatments administered in routine conditions and support changes to labels (eg, new drug indications, new efficacy claims, addition of target populations, addition of administration information and patient‐preference data, safety revisions, and additional information on health‐related outcomes).^22^, ^23^ Owing to the longer duration, real‐world data collection has the potential to demonstrate treatment effects that may not be realized until later in the disease course, beyond the period of observation in a clinical trial.^1^, ^18^ Furthermore, observational studies may provide opportunities to evaluate larger, more diverse, and less restricted populations compared with clinical trials, with exclusion criteria generally based on safety rather than selecting a homogeneous sample.^1^, ^17^, ^22^

Obtaining RWE requires efficient collection and robust analysis of data across disparate centers (eg, implementing common assessment tools, data quality systems, data exchange, data analysis, pooling of data, and strategies for interpretation across clinics).^8^ Gathering data and comparing outcomes of patients with confirmed AD in routine clinical practice can be challenging because cognitive, functional, neuropsychiatric, and quality of life (QoL) outcomes may not be measured consistently.^22^ There is also a lack of consensus on optimal clinical assessments for longitudinal observational studies across the disease continuum from mild cognitive impairment to dementia, with global and regional variations in the instruments used at each disease stage, as well as a lack of large historical longitudinal datasets in patients with confirmed anti‐Aβ pathology. Finally, a variety of different healthcare professionals manage patients with AD, including advanced practice providers, family medicine practitioners, internal medicine specialists, neurologists, medical directors of residential facilities, and geriatric psychiatrists, adding further complexity to the issue. As a result of these various factors, RWE in AD can be difficult to interpret across cohorts and has poor transferability across healthcare systems.^22^

Given these challenges, a global International Collaboration for Real‐world Evidence in Alzheimer's Disease (ICARE AD) program was created to provide a structure for collecting standardized longitudinal data from eligible patients treated with aducanumab‐avwa in postapproval effectiveness studies. The program will no longer proceed owing to the national policy for coverage released by the CMS stating that anti‐Aβ monoclonal antibodies approved by the FDA through the accelerated approval pathway for AD will be covered by Medicare only for those enrolled in FDA‐ or National Institutes of Health‐approved trials conducted under an investigational new drug application,^24^ thereby limiting the use in clinical practice. Despite this, and in light of the CMS confirming that broader coverage is now available for lecanemab following traditional FDA approval,^25^ the authors of this paper believe the approach to and rationale behind the design of this program offers valuable learnings for the field as robust RWE in AD remains an unmet need. In addition, this proposed program would augment the current CMS National Patient Registry study as it has a longer follow‐up period (5 years vs 2 years) and collects additional data, such as QoL, lifestyle, and biomarker (for future analysis) data.^9^ Data from this program would also complement data derived from the ALZ‐NET registry, which is a general database that collects data from patients receiving any FDA‐approved drug for AD, not specifically anti‐Aβ monoclonal antibodies, and does not collect QoL data.^26^ Here we provide considerations for future studies assessing the use of anti‐Aβ monoclonal antibodies in a real‐world setting, with a structure to utilize predefined, consistent assessments that will provide harmonized effectiveness and safety data and patient‐ and caregiver‐reported outcomes.

## 2 STUDY AIMS

Long‐term clinical, QoL, and safety outcomes, including the incidence of ARIA, will be important to measure in future real‐world AD studies. In addition, tools that provide insights into healthcare resource utilization and AD‐related burden on informants/caregivers are valuable. Given an increasing emphasis placed on biomarkers, the collection of blood (plasma and serum) and optional cerebrospinal fluid for biobanking and potential genetic analysis is encouraged. Collectively, such resources and data could serve as a platform for nested studies aiming to support progress in specific areas of interest related to digital assessments, magnetic resonance imaging and ARIA monitoring, and use of new blood‐based biomarkers.

**RESEARCH IN CONTEXT**

1. **Systematic review**: A literature review highlighted that obtaining and comparing real‐world outcomes for patients with Alzheimer's disease (AD) is difficult owing to variation in data collected across centers.
2. **Interpretation**: There is a need to examine the long‐term, real‐world effectiveness and safety of therapies targeting known pathological processes of AD. We provide considerations for observational studies aiming to provide consistent longitudinal data from patients with AD, which will allow comparison of data across regions and transferability across healthcare systems to maximize data interpretability and inform best practices. Collecting data over a longer period and treating a more diverse population compared with registrational Phase III clinical trials will improve generalizability of findings.
3. **Future directions**: This paper aims to provide a framework for consistent data collection in real‐world AD registries and studies as additional treatments enter clinical practice.

## 3 STUDY POPULATIONS

The suggested eligibility criteria in Table 1 allow assessment of a larger and more heterogeneous patient population than is typically included in clinical trials, especially those who are historically underrepresented (eg, individuals who are ethnically/racially/geographically/socioeconomically diverse, have comorbid conditions or atypical disease, or are taking concomitant medications) to ensure that robust data are collected.^1^, ^17^, ^19^, ^22^ Expanded study eligibility can also enhance participant enrollment and retention, further driving patient diversity,^27^ and ensure that study populations are representative of real‐world patients.^28^, ^29^

**TABLE 1:** Suggested eligibility criteria.

Standard inclusion criteria
Patients aged ≥18 years Diagnosis of AD (using investigator's choice of confirmatory assessments consistent with Appropriate Use Recommendations^14^) Informed consent for data collection and able to complete QoL questionnaires Requirement of an informant or caregiver who, in the investigator's opinion, has sufficient contact with the patient to provide accurate information about the patient's cognitive, functional, and behavioral status Receiving anti‐Aβ monoclonal antibody treatment

Standard exclusion criteria
Individuals unable to provide informed consent or to comply with study requirements, including blood (plasma and serum) collection and biobanking Concurrently participating in any interventional clinical studies (participation in other noninterventional studies is permitted) Treated with any investigational drug within 30 days or five half‐lives (whichever is longer) prior to enrollment Pregnant/breastfeeding Contraindications to anti‐Aβ monoclonal antibody treatment

Abbreviations: Aβ, amyloid beta; AD, Alzheimer's disease; QoL, quality of life.

## 4 STUDY OUTCOMES

Recommendations for primary, secondary, tertiary, and exploratory objectives and corresponding endpoints are described in Table 2.

**TABLE 2:** Proposed study objectives and endpoints.

Primary objective	Primary endpoints
Evaluate long‐term AD progression in treated patients with early‐stage AD (MCI due to AD or mild AD dementia) as determined by cognitive, functional, and neuropsychiatric measures and to describe the QoL, caregiver burden, and HCRU longitudinally	Changes in cognition, functional abilities, and neuropsychiatric status as measured during routine visits from baseline through end of study by: QDRS‐IV QDRS‐PV A‐IADL‐Q‐SV FAQ MoCA (version 8.1) NPI‐Q GDS‐SF Changes in QoL, caregiver burden, and HCRU captured during routine visits from baseline through end of study by: 13‐item QoL‐AD ZBI (22 items) SF‐12 (version 2) RUD Lite PGI‐S PGI‐C

Secondary objectives	Secondary endpoints
Evaluate incidence of AEs, including SAEs, in treated patients	Incidence of AEs including SAEs in treated patients
Assess incidence of treatment‐associated ARIA in real‐world practice per label recommendation for safety monitoring Estimate incidence of symptomatic ARIA in real‐world clinical practice Compare incidence of SAEs in patients with and without ARIA	Incidence of ARIA‐E and ARIA‐H (micro‐hemorrhage and superficial siderosis) in treated patients Incidence of symptomatic ARIA and type of symptoms of ARIA (AEs deemed related to ARIA as determined by investigator) Incidence and type of SAEs in treated patients with and without ARIA
Evaluate incidence of brain hemorrhage‐treated patients in real‐world clinical practice	Incidence and clinical symptoms of brain hemorrhages
Obtain descriptive statistics on characteristics of treatment population and drug utilization	Descriptive statistics on patient characteristics, including age, sex, geography, comorbidities, lifestyle, concomitant AD and other medications, and stage and severity of AD at drug initiation (including any off‐label use) Descriptive statistics on frequency of prescription; descriptive statistics on ARIA‐E monitoring patterns in relation to approved label Descriptive statistics on treatment duration and incidence of discontinuation

**Tertiary objective**	**Tertiary endpoint**
Generate longitudinal biobank for future AD plasma or serum biomarker research	Blood samples (plasma and serum) throughout duration of patient participation in study at baseline and at approximately Months 6, 12, 24, 36, 48, and 60

Exploratory objectives (where available)	Exploratory endpoints
Evaluate long‐term treatment effects on biomarkers related to AD development or progression Collect samples for future genetic and biomarker research	Optional CSF sample with biomarker data: change in levels of fluid biomarkers related to disease, which may include, but are not limited to, amyloid and tau proteins Optional, one‐time collection of blood for future genetic and biomarker research

Abbreviations: AD, Alzheimer's disease; AE, adverse event; A‐IADL‐Q‐SV, Amsterdam Instrumental Activities of Daily Living Questionnaire Short Version; ARIA, amyloid‐related imaging abnormalities; ARIA‐E, amyloid‐related imaging abnormalities due to vasogenic edema; ARIA‐H, amyloid‐related imaging abnormalities due to micro‐hemorrhages, macro‐hemorrhages; CSF, cerebrospinal fluid; FAQ, Functional Activities Questionnaire; GDS‐SF, Geriatric Depression Scale Short Form; HCRU, healthcare resource utilization; MoCA, Montreal Cognitive Assessment; NPI‐Q, Neuropsychiatric Inventory Questionnaire; PGI‐C, Patient Global Impression of Change; PGI‐S, Patient Global Impression of Severity; QDRS‐IV, Quick Dementia Rating System Informant Version; QDRS‐PV, Quick Dementia Rating System Patient Version; QoL, quality of life; QoL‐AD, Quality of Life in Alzheimer's Disease; RUD, Resource Utilization in Dementia; SAE, serious adverse event; SF‐12, 12‐item Short Form Survey; ZBI, Zarit Burden Interview.

The recommended assessment tools were selected in collaboration with an external steering committee of international clinical experts and patient advisory groups, following a review and analysis of instruments utilized in cohorts and registries. The criteria for instrument selection considered sensitivity to assess changes in cognition, functional abilities, and neuropsychiatric status in early‐stage AD (mild cognitive impairment due to AD and mild AD dementia), which were identified through a comprehensive literature search of research analyzing test sensitivity. Further criteria included reliability, validity, ability to comprehensively assess AD clinical manifestations, and availability of existing data for untreated patients. Additionally, the measures need to be feasible for use in routine specialist practice, with appropriate formats for diverse populations, and easier and more rapid administration relative to clinical trial measures, facilitating efficient incorporation into the patient management workflow. Collection of descriptive information on the characteristics of patients with AD was recommended to gain a better understanding of AD, and the inclusion of QoL and health economic measures was intended to support measurement of the benefit/risk profile of future anti‐Aβ monoclonal antibodies entering clinical practice. Further details regarding the rationale for including specific assessment tools are provided in Table 3. An overview of the administration, validity, and limitations of the instruments is given in Data S1.

**TABLE 3:** Rationale for suggested assessment tools.

Assessment tool	Rationale for inclusion
Montreal Cognitive Assessment (MoCA)	Brief and easy‐to‐administer screening tool assessing cognition that is available in multiple languages^44^, ^45^ Allows for simple adjustment of education level (one point added to total score for patients with ≤12 years of education)^44^ Can be administered remotely by trained clinicians to increase access and ease of use (audiovisual conferencing and abbreviated telephone versions are available)^46^ Involves more words, potentially fewer learning trials, and a longer delay before recall compared with MMSE^44^ Better assesses cognitive domains of executive functions and working memory, which are important domains to evaluate, given that they are often impaired in early‐stage AD,^44^ compared with MMSE
Quick Dementia Rating System Informant Version/Patient Version (QDRS‐IV/PV)	Rapid informant‐ and patient‐based screening tools assessing cognition^47^, ^48^ Able to differentiate individuals with and without cognitive impairment and provide accurate dementia staging^47^, ^48^ No extensive training or clinician input required and can be completed prior to a physician visit^47^, ^48^ The patient version can be used when caregivers are not available^48^ Strongly correlated with gold‐standard evaluations, such as CDR‐G and CDR‐SB, neuropsychological testing, and other validated measures of dementia severity, behavior, and caregiver concerns^47^, ^48^ Validity has been demonstrated in research and community real‐world settings and demonstrates good correlation with AD biomarkers^47^, ^49^
Amsterdam Instrumental Activities of Daily Living Questionnaire Short Version (A‐IADL‐Q‐SV)	A computerized questionnaire assessing functional abilities, specifically day‐to‐day problems caused by cognitive difficulties^50^ Comparable psychometric properties with A‐IADL‐Q, but shorter length makes it more suitable for everyday clinical practice^50^ Developed with input from patients, caregivers, and healthcare providers, supporting content validity^51^ High construct validity, supported by concordance with MMSE^50^ and limited correlation with age, sex, and education^52^ High test‐retest reliability, very high internal consistency, and unidimensional factor structure^50^ Able to differentiate between various diagnostic groups with respect to IADL impairment^50^ High sensitivity to change compared with traditional IADL outcomes^53^ Related to AD‐specific neurodegeneration^54^ Completed by study partner, with a self‐report version also available^55^ Extensive cross‐cultural validation supports use of different languages and culturally adapted versions^56^ Cut‐off values available for clinically meaningful decline, as determined across stages of AD in mixed‐methods study^57^
Neuropsychiatric Inventory Questionnaire (NPI‐Q)	A short screening questionnaire to assess neuropsychiatric symptoms and the associated impact on caregivers^58^, ^59^ A self‐administered questionnaire completed by informants about patients under their care, which provides useful information when working with patients’ caregivers and families^58^, ^59^ Most caregivers complete questionnaire in less than 5 min, meaning it is feasible to implement in routine clinical practice^58^, ^59^ Adequate test‐retest reliability^58^ The total symptom score is robustly correlated with the NPI in populations with a range of dementia severities^58^
Quality of Life in Alzheimer's Disease (QoL‐AD)	A brief, easily administered assessment of QoL in AD^60^ Rates patient's QoL from both patient and caregiver via interview and self‐completed questionnaire, respectively. The two scores can be considered individually, or a composite score can be calculated^60^, ^61^ Uses simple/straightforward language and responses to optimize use in patients with cognitive impairment^60^, ^61^ Reliably and validly rates QoL of a patient with mild to moderate dementia^60^, ^61^
12‐item Short‐Form Survey (SF‐12)	A widely used instrument for assessing self‐reported health‐related QoL^62^, ^63^ Takes less than 2 min to complete, making it a useful tool for those with limited attention spans^62^ Includes the same eight health domains as the SF‐36 with substantially fewer questions^62^, ^63^ A high degree of correlation with SF‐36^62^ Proven to be reliable and valid in clinical and population‐based applications^62^
Patient Global Impression of Severity/Change (PGI‐S/C)	Quick assessments collecting data on patients’ perceptions of their condition^64^ Simple to use, scores are easily calculated, and no training is required for their use^64^ Correlates moderately to highly with other patient‐reported measures, including across variations in ethnicity^65^
Zarit Burden Interview (ZBI)	A commonly used instrument providing a comprehensive assessment of objective and subjective burden for caregivers of patients with dementia^66^ Validated in many culturally and ethnically diverse populations^66^ Highly correlated with other standardized instruments^66^
Resource Utilization in Dementia (RUD) Lite Scale	Assesses both formal and informal resource use, meaning it is possible to calculate costs from a societal perspective^67^ Demonstrated to be a valid measure of the amount of time provided by caregivers to patients with dementia^67^

Abbreviations: AD, Alzheimer's disease; A‐IADL‐Q, Amsterdam Instrumental Activities of Daily Living Questionnaire; A‐IADL‐Q‐SV, Amsterdam Instrumental Activities of Daily Living Questionnaire Short Version; CDR‐G, Clinical Dementia Rating Global Score; CDR‐SB, Clinical Dementia Rating Scale‐Sum of Boxes; IADL, Instrumental Activities of Daily Living; MMSE, Mini‐Mental State Examination; MoCA, Montreal Cognitive Assessment; NPI, Neuropsychiatric Inventory; NPI‐Q, Neuropsychiatric Inventory Questionnaire; PGI‐C, Patient Global Impression of Change; PGI‐S, Patient Global Impression of Severity; QDRS‐IV, Quick Dementia Rating System Informant Version; QDRS‐PV, Quick Dementia Rating System Patient Version; QoL, quality of life; QOL‐AD, Quality of Life in Alzheimer's Disease; RUD, Resource Utilization in Dementia; SF‐12, 12‐item Short‐Form Survey; SF‐36, 36‐item Short Form Survey; ZBI, Zarit Burden Interview.

Data collected as part of a real‐world study may be compared with matched external control groups (ie, from existing AD cohorts that represent comparators for natural disease progression, historical health insurance claims, or future registries) to characterize real‐world, long‐term changes in cognition, functional abilities, and neuropsychiatric status and evaluate any differences between treated and untreated patients and differences among treatments.^30^ RWE may also be compared with data from placebo arms of clinical trials while acknowledging the limitations of existing AD trial cohorts (such as lack of diversity) when interpreting comparisons with these groups.^19^, ^21^ In this proposed study, including patients eligible for treatment but who decline this could be considered, but patients who decline treatment may be difficult to follow up with, which could result in a misleading and partial dataset for such a comparator group.

In addition to measuring clinical outcomes, collecting blood and cerebrospinal fluid specimens is recommended for future biomarker and genetic studies in AD. Recent data have highlighted potential future applications of biomarkers, including as measures of disease progression, as prescreening tools for identifying patients with Aβ pathology and other AD‐related pathologies, and as screening tools for diagnosis and prognosis of patients to be included in clinical trials.^31^, ^32^, ^33^, ^34^, ^35^, ^36^, ^37^, ^38^, ^39^ Biomarkers may also have a role in monitoring treatment response by demonstrating target engagement or as a surrogate of clinical benefit, as well as potentially predicting and monitoring side effects, such as ARIA due to vasogenic edema.^32^, ^39^, ^40^, ^41^, ^42^ Biomarkers are now also being used to inform treatment decisions in clinical practice, with the aducanumab‐avwa and lecanemab‐irmb PIs stating that the presence of Aβ pathology must be confirmed prior to initiating treatment.^5^, ^6^ However, there remain a lack of consensus on the choice of biomarker assays and cutoffs, an absence of prospectively collected data, and missing validation of key assay performance characteristics, especially in real‐world populations.^31^, ^39^, ^43^ Biobanking of longitudinal samples could enable biomarker substudies to be conducted and generate evidence for the validation, regulatory approval, and adoption of biomarkers as measures of brain AD pathology in patients with cognitive impairment, disease progression, and treatment response. Standardized procedures for sample collection and specimen storage need to be provided by the sponsors of the study.

## 5 DATA COLLECTION

Study durations of at least 5 years are recommended to ensure adequate observation of longitudinal changes, given that Aβ plaque levels have been found to continue to decline beyond 4 years of treatment with some anti‐Aβ monoclonal antibodies.^5^

To improve the diversity of enrolled participants, the FDA recommends accounting for logistical and patient‐related factors that may limit participation in clinical studies, for example, reducing the burden of study participation by minimizing study visits and employing enrollment practices that enhance inclusiveness.^29^ As such, study sites should be selected to represent a range of geographical locations and AD care settings (eg, academic centers, community memory clinics) with ethnically, racially, and socioeconomically diverse populations, as well as consideration of their ability to implement the core data elements. Data should typically be collected by trained site personnel as part of a structured interview (clinical assessment tools) or collected prospectively as part of an unstructured interview (medical history). For a real‐world study of this duration, it is recommended that data be gathered during routine visits conducted within the context of standard of care, with intervals for data collection of approximately 6 to 12 months. Self‐administered cognitive assessments, such as the Quick Dementia Rating System Patient Version (QDRS‐PV), which can be completed prior to the physician visit, may reduce study site burden, but they can be associated with some challenges, including reduced response validity (compliance, effort, motivation) because of a lack of monitoring, loss of qualitative data obtained from conventional evaluations, risk of interruptions, and lack of support for the patient if they are struggling to complete the task or experience technological problems.^48^, ^68^

**FIGURE 1:**
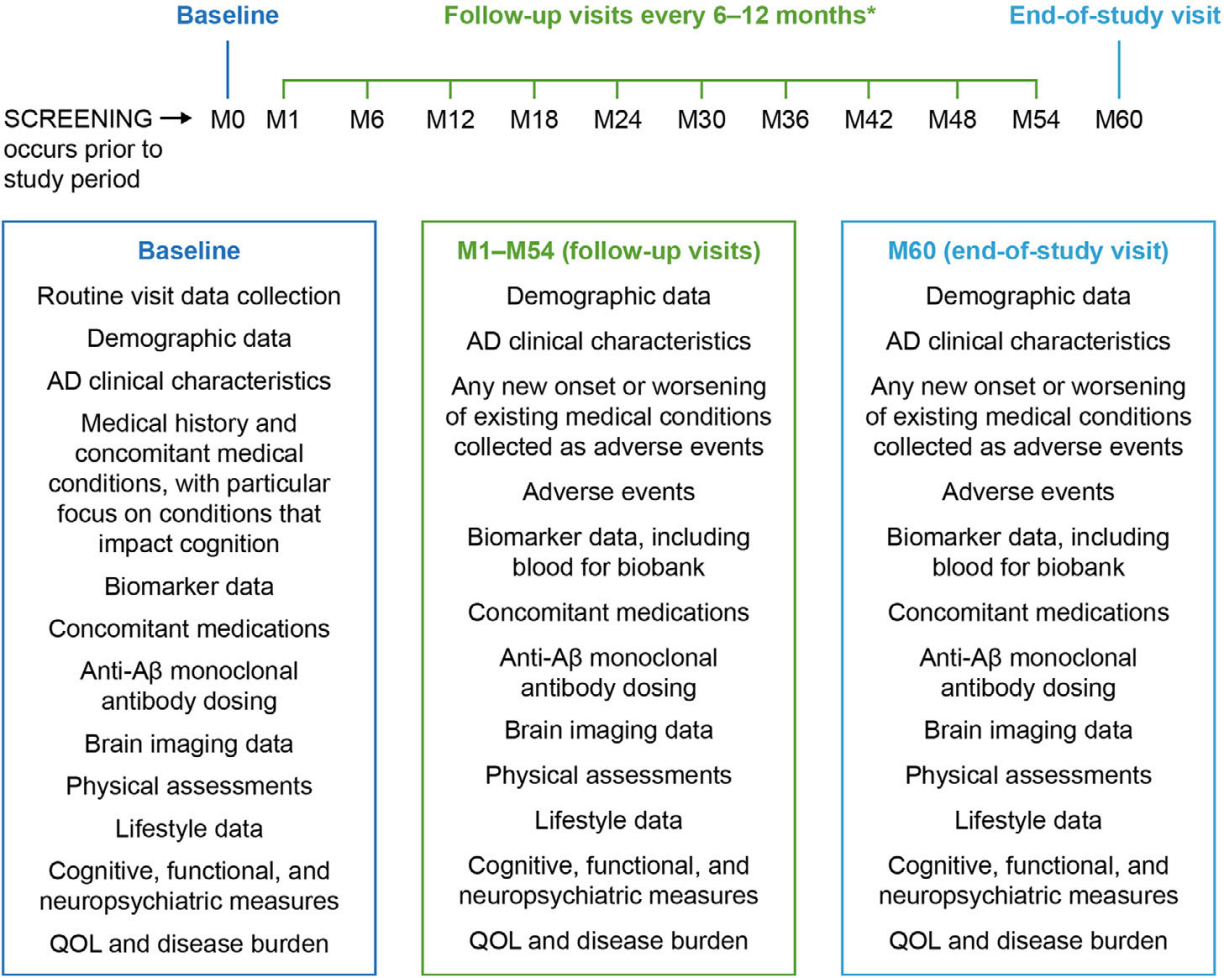
Schedule of data collection. *Study information may be collected at any routine clinical visits scheduled per local standard of care. Data from radiological assessments of MRI scans will be collected at each MRI visit. It is expected that after a patient reaches target dosing, information on each patient will be collected at the physician's discretion or as part of routine clinical practice, anticipated to be approximately every 6 to 12 months. AD, Alzheimer's disease; M, month; MRI, magnetic resonance imaging; QoL, quality of life.

An overview of the recommended schedule of data collection is shown in Figure 1. A detailed schedule of assessments is listed in Table S1; core data elements are noted to ensure consistency of clinical assessments and demographics collection, with data that may not be collected in routine practice considered optional to minimize study site burden and maximize real‐world relevance.[Table alz13785-tbl-0004]

## 6 LIMITATIONS OF PROPOSED REAL‐WORLD STUDIES

The potential limitations of a real‐world study are that some components may not be typical of clinical practice, namely, trained raters, extensive data and biomarker collection, and the requirement to enter results into a database, all of which may result in greater recruitment from research centers than other clinical settings. Moreover, the practicalities of implementing such a core dataset may be challenging, owing to regional variations in resource availability, differences in clinician specialty and training, the requirement for translation and validation of assessment tools, and reimbursement of AD biomarker investigations. The process of recruitment and consent in such a study may also introduce bias based on comorbidities, language, socioeconomic status, and ethnicity.

## 7 CONCLUSION

An important objective of the current AD treatment landscape is to monitor patient outcomes and evaluate disease progression. Real‐world studies can be compromised by inconsistent collection of data and use of nonstandardized endpoints, which can limit the generalizability of results. As summarized in Table 4, these considerations for collecting RWE in AD, adapted from the now closed ICARE AD program, may address some of the limitations of RCTs and help ensure consistent, longitudinal data collection across the AD continuum in routine practice to allow for transferability of data across healthcare systems.

**TABLE 4 alz13785-tbl-0004:** Summary of proposed RWE benefits.

Limitations of RCTs	Benefits of proposed RWE
Population diversity	Broader eligibility criteria; inclusion of community healthcare settings; minimal trial participant burden; inclusion of patients underrepresented in clinical trials
Study duration	Longer period of observation to evaluate longitudinal changes
Generalizability and transferability of data	Robust framework for collecting harmonized data, with core elements across all participating centers for consistent collection and interpretation; consensus on optimal clinical assessment tools
Selectivity and sensitivity of instruments	Range of well‐validated measures of cognition, functional abilities, neuropsychiatric status, quality of life, health economics, safety, and drug utilization
Healthcare‐related burden evaluation	Inclusion of patient‐ and caregiver‐reported outcomes, QoL, and HCRU measures in real‐world clinical context
Real‐world applicability	Clinical assessments tailored to standard‐of‐care clinical practice in varied healthcare settings
Data interpretation	Comparison with matched natural history cohorts, health insurance claims, or registries to evaluate differences between treated and untreated patients

Abbreviations: HCRU, healthcare resource utilization; QoL, quality of life; RCT, randomized controlled trial; RWE, real‐world evidence.

## AUTHOR CONTRIBUTIONS

All authors participated in the review of the literature and the drafting and review of the manuscript. All authors read and approved the final manuscript.

## CONFLICT OF INTEREST STATEMENT

J.E.G.: Provided consultation to Alpha Cognition, Biogen, Cognition Therapeutics, CND Life Sciences, EIP Pharma, Eisai, Eli Lilly, GE Healthcare, Genentech, Otsuka, and Roche. J.E.G. is the Chief Scientific Officer for Cognivue, the creator of the Quick Dementia Rating System, and holds the copyright with the New York University Grossman School of Medicine. J.E.G. is supported by National Institute on Aging (NIA) grants R01AG071514, R01AG701514S1, R56AG074889, R01AG071643, R01AG069765, R01AG057681, P01AG066584, and P30AG059295 and National Institute of Neurological Disorders and Stroke (NINDS) grants R01NS101483 and R01NS101483S1. J.L.C.: Provided consultation to Acadia, Actinogen, Acumen, AlphaCognition, Aprinoia, AriBio, Artery, Biogen, BioVie, Cassava, Cerecin, Diadem, EIP Pharma, Eisai, GemVax, Genentech, GAP Innovations, Janssen, Jocasta, Karuna, Lilly, Lundbeck, LSP, Merck, NervGen, Novo Nordisk, Oligomerix, Ono, Optoceutics, Otsuka, PRODEO, Prothena, ReMYND, Roche, Sage Therapeutics, Signant Health, Simcere, Sunbird Bio, Suven, SynapseBio, TrueBinding, Vaxxinity, and Wren Therapeutics pharmaceutical, assessment, and investment companies. J.L.C. is supported by National Institute of General Medical Sciences grant P20GM109025; NINDS grant U01NS093334; NIA grants R01AG053798, P20AG068053, P30AG072959, and R35AG71476; Alzheimer's Disease Drug Discovery Foundation (ADDF); Ted and Maria Quirk Endowment; and the Joy Chambers‐Grundy Endowment. M.L.B., C.deM., C.M.G., and M.R.S.: During the development of the study protocol and development of the manuscript, they were employees of Biogen and may hold stock, but have since left the company. I.R. is an employee of Biogen and may hold stock. R.F.A.: Supported by grants from Alzheimer's Association, CONICET (Consejo Nacional de Investigaciones Científicas y Tecnológicas de Argentina), Fleni Foundation, and subcontract funding from NIH (R01AGO53267). R.F.A. has served as a consultant or principal investigator for Bago, Biogen, Merck, Novo‐Nordisk, and Roche. A.A.: Received honoraria for consulting, participated in independent data safety monitoring boards, provided educational lectures, programs, and materials, and served on advisory boards for AbbVie, Acadia, Allergan, Alzheimer's Association, Alzheimer's Disease International (ADI), Axovant, AZ Therapies, Biogen, Eisai, Grifols, Harvard Medical School Graduate Continuing Education, JOMDD, Lundbeck, Merck, Prothena, Roche/Genentech, Novo Nordisk, Qynapse, Sunovion, Suven, and Synexus. A.A. receives royalties from Oxford University Press for a medical book on dementia and has received institutional grants and contract funding from NIA/NIH 1P30AG072980, AZ DHS CTR040636, Washington University St. Louis, Foundation for NIH (FNIH), and Gates Ventures. A. Atri's institution receives funding for clinical trials, biomarker and observational studies, contracts, and projects from AD consortia, foundations, and companies for which A.A. serves as the site principal investigator (past institution received funding for the Biogen EMERGE study; current institution receiving funding for the Eisai‐sponsored AHEAD 3‐45 study). H.C.: Supported by a foundation grant from Canadian Institutes for Health Research (CIHR), along with the Weston Foundation and the Baycrest Health Sciences Foundation. In the past 5 years, H.C. has participated as a site principal investigator in pharmaceutical trial activities sponsored by Alector LLC, Anavex Life Sciences, Hoffmann‐La Roche Limited, Lilly, TauRx, and Immunocal (site investigator for trials). H.C. participated as an unpaid advisor in 2020/21 for the establishment of an international database by Biogen (ICARE AD). C.P.: Member of the International Advisory Boards of Lilly and a consultant for Ads Neuroscience, AgenT, Alzhois, Euroimmune, Fujirebio, Roche, and Gilead. C.P. is an investigator in several clinical trials for AstraZeneca, Biogen, Esai, Lilly, Lundbeck, Neuroimmune, and Roche. V.R.P.: Provided consultation to Biogen and is supported by grants from the National Institutes of Health, Providence St. Joseph Health (Alzheimer's Translational Pillar [ATP]); Pacific Neuroscience Institute Foundation, including the generous support of the Singleton and McLoughlin families, and Saint John's Health Center Foundation. C.W.R.: Provided consultation to AbbVie, Actinogen, Alchemab Therapeutics, Biogen, Brain Health Scotland, Eisai, Lilly, Merck, Novo Nordisk, Roche, Roche Diagnostics, Signant Health, and Sygnature Discovery and is supported by grants from Biogen, AC Immune SA, and Roche. C.W.R. has intellectual property developed at the University of Edinburgh licensed to Linus Health and is Chief Executive Officer and Founder of Scottish Brain Sciences. S.A.M.S.: Supported by grants from Health∼Holland, Top sector Life Sciences & Health (PPP‐allowance; nos. LSHM20084 and LSHM19051), and ZonMW (nos. 7330502051 and 73305095008). Served as a consultant for Boehringer Ingelheim, Lundbeck, Takeda, and Toyama. S.A.M.S. is the developer and receives license fees for the use of the Amsterdam IADL Questionnaire from Alzheon, Axon Neuroscience, Genentech, Green Valley, Janssen, MedAvante, Roche, Vivoryon, and vTv Therapeutics. All funding, consultancy fees, and license fees are paid to S.A.M. Sikkes’ institution. Author disclosures are available in the supporting information.

## Supporting information

Supporting Information

Table S1

ICMJE Disclosure Form

## References

[1] Nazha B , Yang JC‐H , Owonikoko TK . Benefits and limitations of real‐world evidence: lessons from EGFR mutation‐positive non‐small‐cell lung cancer. Future Oncol. 2021;17:965‐977. doi:10.2217/fon-2020-0951 33242257

[2] U.S. Food & Drug Administration (FDA) . Framework for FDA's real‐world evidence program 2018. (accessed March 8, 2022). https://www.fda.gov/media/120060/download

[3] U.S. Food & Drug Administration (FDA) . Real‐world evidence 2022. (accessed January 27, 2023). https://www.fda.gov/science‐research/science‐and‐research‐special‐topics/real‐world‐evidence

[4] Bonamici S. H.R.34—114th Congress (2015‐2016): 21st Century Cures Act 2016. (accessed March 22, 2023). https://www.congress.gov/bill/114th‐congress/house‐bill/34/

[5] U.S. Food & Drug Administration (FDA) . ADUHELM: highlights of prescribing information (PI) 2023. (accessed March 19, 2023). https://www.accessdata.fda.gov/drugsatfda_docs/label/2023/761178s007lbl.pdf

[6] U.S. Food & Drug Administration (FDA) . LEQEMBI: highlights of prescribing information (PI) 2023. (accessed October 5, 2023). https://www.accessdata.fda.gov/drugsatfda_docs/label/2023/761269Orig1s001lbl.pdf

[7] Alzheimer's Network for Treatment and Diagnostics. ALZ‐NET 2023. (accessed March 19, 2023). https://www.alz‐net.org/

[8] European Medicines Agency (EMA) . Guideline on registry‐based studies 2020. (accessed October 16, 2020). https://www.ema.europa.eu/en/documents/scientific‐guideline/guideline‐registry‐based‐studies_en.pdf

[9] Centers for Medicare and Medicaid Services (CMS) . Prospective study on anti‐amyloid‐β monoclonal antibodies directed against amyloid for the treatment of Alzheimer's disease coverage of evidence development (the Anti‐aβ mAb CED study) 2023. (accessed October 5, 2023). https://www.cms.gov/files/document/ced‐study‐description.pdf

[10] Yang W , Zilov A , Soewondo P , Bech OM , Sekkal F , Home PD . Observational studies: going beyond the boundaries of randomized controlled trials. Diabetes Res Clin Pract. 2010;88(1):S3‐S9. doi:10.1016/S0168-8227(10)70002-4 20466165

[11] Ismail RK , Sikkes NO , Wouters MW , et al. Postapproval trials versus patient registries: comparability of advanced melanoma patients with brain metastases. Melanoma Res. 2021;31:58‐66. doi:10.1097/CMR.0000000000000707 33351553 PMC7757745

[12] U.S. Food & Drug Administration (FDA) . FDA converts novel Alzheimer's disease treatment to traditional approval 2023. (accessed October 5, 2023). https://www.fda.gov/news‐events/press‐announcements/fda‐converts‐novel‐alzheimers‐disease‐treatment‐traditional‐approval

[13] U.S. Food & Drug Administration (FDA) . FDA grants accelerated approval for Alzheimer's drug 2021. (accessed October 5, 2023). https://www.fda.gov/news‐events/press‐announcements/fda‐grants‐accelerated‐approval‐alzheimers‐drug

[14] Cummings J , Rabinovici GD , Atri A , et al. Aducanumab: Appropriate Use Recommendations update. J Prev Alzheimers Dis. 2022;9:221‐230. doi:10.14283/jpad.2022.34 35542993 PMC9169517

[15] Cummings J , Apostolova L , Rabinovici GD , et al. Lecanemab: Appropriate Use Recommendations. J Prev Alzheimers Dis. 2023;10:362‐377. doi:10.14283/jpad.2023.30 37357276 PMC10313141

[16] Spieth PM , Kubasch AS , Penzlin AI , Illigens BM‐W , Barlinn K , Siepmann T . Randomized controlled trials—a matter of design. Neuropsychiatr Dis Treat. 2016;12:1341‐1349. doi:10.2147/NDT.S101938 27354804 PMC4910682

[17] Depp C , Lebowitz BD . Clinical trials: bridging the gap between efficacy and effectiveness. Int Rev Psychiatry. 2007;19:531‐539. doi:10.1080/09540260701563320 17896233

[18] Atri A , Rountree SD , Lopez OL , Doody RS . Validity, significance, strengths, limitations, and evidentiary value of real‐world clinical data for combination therapy in Alzheimer's disease: comparison of efficacy and effectiveness studies. Neurodegener Dis. 2012;10:170‐174. doi:10.1159/000335156 22327239 PMC3702018

[19] Banzi R , Camaioni P , Tettamanti M , Bertele’ V , Lucca U . Older patients are still under‐represented in clinical trials of Alzheimer's disease. Alzheimers Res Ther. 2016;8:32. doi:10.1186/s13195-016-0201-2 27520290 PMC4982205

[20] Mitchell AK , Massett HA , Shakur M , Lockett J , Han SH . Analysis of exclusion criteria in NIA‐funded Alzheimer's disease and Alzheimer's disease‐related dementias clinical trials. Alzheimers Dement. 2021;17(10):e054416. doi:10.1002/alz.054416

[21] Franzen S , Smith JE , van den Berg E , et al. Diversity in Alzheimer's disease drug trials: the importance of eligibility criteria. Alzheimers Dement. 2022;18:810‐823. doi:10.1002/alz.12433 34590409 PMC8964823

[22] Gallacher J , de Reydet de Vulpillieres F , Amzal B , et al. Challenges for optimizing real‐world evidence in Alzheimer's disease: the ROADMAP project. J Alzheimers Dis. 2019;67:495‐501. doi:10.3233/JAD-180370 30584137 PMC6398537

[23] Tashkin DP , Amin AN , Kerwin EM . Comparing randomized controlled trials and real‐world studies in chronic obstructive pulmonary disease pharmacotherapy. Int J Chron Obstruct Pulmon Dis. 2020;15:1225‐1243. doi:10.2147/COPD.S244942 32581529 PMC7276323

[24] Hoffman M . Biogen terminates Phase 4 ICARE AD trial of aducanumab in Alzheimer's disease 2022. (accessed July 28, 2022). https://www.neurologylive.com/view/biogen‐terminates‐phase‐4‐icare‐ad‐trial‐aducanumab‐alzheimer‐disease

[25] Centers for Medicare and Medicaid Services (CMS) . Statement: broader Medicare coverage of LEQEMBI available following FDA traditional approval 2023. (accessed October 5, 2023). https://www.cms.gov/newsroom/press‐releases/statement‐broader‐medicare‐coverage‐leqembi‐available‐following‐fda‐traditional‐approval

[26] Alzheimer's Association. ALZ‐NET protocol synopsis 2023. (accessed October 5, 2023). https://www.alznetproviders.org/‐/media/ALZNET/Resources/ALZ‐NET‐Protocol‐Synopsis.pdf

[27] Benbow JH , Rivera DR , Lund JL , Feldman JE , Kim ES . Increasing inclusiveness of patient‐centric clinical evidence generation in oncology: real‐world data and clinical trials. Am Soc Clin Oncol Educ Book. 2022:116‐126. doi:10.1200/EDBK_350574 35561304

[28] U.S. Food & Drug Administration (FDA) . Diversity plans to improve enrollment of participants from underrepresented racial and ethnic populations in clinical trials; draft guidance for industry; availability 2022. (accessed February 6, 2023). https://www.fda.gov/regulatory‐information/search‐fda‐guidance‐documents/diversity‐plans‐improve‐enrollment‐participants‐underrepresented‐racial‐and‐ethnic‐populations

[29] U.S. Food & Drug Administration (FDA) . Enhancing the diversity of clinical trial populations — eligibility criteria, enrollment practices, and trial designs guidance for industry 2020. (accessed February 6, 2023). https://www.fda.gov/regulatory‐information/search‐fda‐guidance‐documents/enhancing‐diversity‐clinical‐trial‐populations‐eligibility‐criteria‐enrollment‐practices‐and‐trial

[30] Liu J , Barrett JS , Leonardi ET , et al. Natural history and real‐world data in rare diseases: applications, limitations, and future perspectives. J Clin Pharmacol. 2022;62(2):S38‐S55. doi:10.1002/jcph.2134 36461748 PMC10107901

[31] Blennow K , Dage J , Bateman R , Hansson O . Recent advances in plasma biomarkers to improve preclinical and prodromal AD trials. Oral presentation at the 14th Clinical Trials on Alzheimer's Disease (CTAD) Annual Meeting, Boston, Massachusetts, USA, November 9‐12, 2021:(Abstract S1).

[32] Sims JR , Lu M , Schade AE , Brooks DA , Mintun MA . TRAILBLAZER‐ALZ: three clinical trials of donanemab in early Alzheimer's disease: plasma P‐tau assays and the initial performance in clinical trials. Oral presentation at the 14th Clinical Trials on Alzheimer's Disease (CTAD) Annual Meeting, Boston, Massachusetts, USA, November 9‐12, 2021:(Abstract S2).

[33] Verbel D , Gee M , Kaplow J , et al. Prediction of brain amyloid pathology using plasma Aβ42/40 ratio measured using the C2N PrecivityADTM test in the Mission AD study samples. Oral presentation at the 14th Clinical Trials on Alzheimer's Disease (CTAD) Annual Meeting, Boston, Massachusetts, USA, November 9‐12, 2021:(Abstract LBR08).

[34] Pereira J , Janelidze S , Smith R , et al. Plasma GFAP is an early marker of Aβ but not tau pathology in Alzheimer's disease. Oral presentation at the 14th Clinical Trials on Alzheimer's Disease (CTAD) Annual Meeting, Boston, Massachusetts, USA, November 9‐12, 2021:(Abstract OC02).

[35] Schindler S , Yarasheski K , West T , et al. Performance of the PrecivityAD™ blood test in detection of brain amyloidosis in cognitively normal and cognitively impaired individuals. Oral presentation at the 14th Clinical Trials on Alzheimer's Disease (CTAD) Annual Meeting, Boston, Massachusetts, USA, November 9‐12, 2021:(Abstract ROC09).

[36] Ashton N , Milà‐Alomà M , Benedet A , et al. Plasma PTau231 as an early marker of amyloid‐β pathology for preclinical Alzheimer's disease trial selection. Oral presentation at the 14th Clinical Trials on Alzheimer's Disease (CTAD) Annual Meeting, Boston, Massachusetts, USA, November 9‐12, 2021:(Abstract LB01).

[37] Piccirella S , Van Neste L , Fowler C , Doecke J , Uberti D , Kinnon P. AlzoSure® Predict, a simple, non‐invasive blood test to predict the early onset of Alzheimer's disease with the ability to identify MCI patients, before the clinical symptoms are identifiable (in the same test) 6 years in advance of clinical diagnosis. Oral presentation at the 14th Clinical Trials on Alzheimer's Disease (CTAD) Annual Meeting, Boston, Massachusetts, USA, November 9‐12, 2021:(Abstract LB03).

[38] Sperling ER , Johnson K , Zhou J , et al. Introduction of plasma biomarker screening for the AHEAD 3‐45 study. Oral presentation at the 14th Clinical Trials on Alzheimer's Disease (CTAD) Annual Meeting, Boston, Massachusetts, USA, November 9‐12, 2021:(Abstract LB04).

[39] Hansson O , Edelmayer RM , Boxer AL , et al. The Alzheimer's Association Appropriate Use Recommendations for blood biomarkers in Alzheimer's disease. Alzheimers Dement. 2022;18:2669‐2686. doi:10.1002/alz.12756 35908251 PMC10087669

[40] Hansson O , Nisenbaum L , Chen T , et al. Dose‐ and time‐dependent changes in plasma p‐tau^181^ in patients treated with aducanumab in the ENGAGE and EMERGE trials. Oral presentation at the 14th Clinical Trials on Alzheimer's Disease (CTAD) Annual Meeting, Boston, Massachusetts, USA, November 9‐12, 2021:(Abstract Late‐breaking Roundtable 8).

[41] Swanson C , Dhadda S , Irizarry M , et al. Lecanemab: an assessment of the clinical effects, the correlation of plasma Aβ42/40 ratio with changes in brain amyloid PET SUVr, and safety from the core and open label extension of the Phase 2 proof‐of‐concept study, BAN2401‐G000‐201, in subjects with early Alzheimer's disease. Oral presentation at the 14th Clinical Trials on Alzheimer's Disease (CTAD) Annual Meeting, Boston, Massachusetts, USA, November 9‐12, 2021:(Abstract Late‐breaking Roundtable 5).

[42] Piazza F , Caminiti SP , Zedde M , et al. Association of microglial activation with spontaneous ARIA‐E and CSF levels of anti‐aβ autoantibodies. Neurology. 2022;99:e1265‐e1277. doi:10.1212/WNL.0000000000200892 35940900 PMC9576297

[43] Rabe C , Bittner T , Jethwa A , et al. Utility of plasma Aβ1‐42/Aβ1‐40 as a screening tool is limited due to lack of robustness. Oral presentation at the 14th Clinical Trials on Alzheimer's Disease (CTAD) Annual Meeting, Boston, Massachusetts, USA, November 9‐12, 2021:(Abstract LBR11).

[44] Nasreddine ZS , Phillips NA , Bédirian V , et al. The Montreal Cognitive Assessment, MoCA: a brief screening tool for mild cognitive impairment. J Am Geriatr Soc. 2005;53:695‐699. doi:10.1111/j.1532-5415.2005.53221.x 15817019

[45] O'Driscoll C , Shaikh M . Cross‐cultural applicability of the Montreal Cognitive Assessment (MoCA): a systematic review. J Alzheimers Dis. 2017;58:789‐801. doi:10.3233/JAD-161042 28482634

[46] Nasreddine ZS , MoCA test FAQ 2019. (accessed October 9, 2020). https://www.mocatest.org/faq/

[47] Galvin JE . The Quick Dementia Rating System (QDRS): a rapid dementia staging tool. Alzheimers Dement. 2015;1:249‐259. doi:10.1016/j.dadm.2015.03.003 PMC4484882 26140284

[48] Galvin JE , Tolea MI , Chrisphonte S . Using a patient‐reported outcome to improve detection of cognitive impairment and dementia: the patient version of the Quick Dementia Rating System (QDRS). PLoS One. 2020;15:e0240422. doi:10.1371/journal.pone.0240422 33057404 PMC7561106

[49] Duff K , Wan L , Levine DA , et al. The Quick Dementia Rating System and its relationship to biomarkers of Alzheimer's disease and neuropsychological performance. Dement Geriatr Cogn Disord. 2022;51:214‐220. doi:10.1159/000524548 35477163 PMC9357090

[50] Jutten RJ , Peeters CF , Leijdesdorff SM , et al. Detecting functional decline from normal aging to dementia: development and validation of a short version of the Amsterdam IADL Questionnaire. Alzheimers Dement (Amst). 2017;8:26‐35. doi:10.1016/j.dadm.2017.03.002 28462387 PMC5403784

[51] Sikkes SAM , de Lange‐de Klerk ESM , Pijnenburg YAL , et al. A new informant‐based questionnaire for instrumental activities of daily living in dementia. Alzheimers Dement. 2012;8:536‐543. doi:10.1016/j.jalz.2011.08.006 23102123

[52] Sikkes SA , Knol DL , Pijnenburg YA , de Lange‐de Klerk ES , Uitdehaag BM , Scheltens P . Validation of the Amsterdam IADL Questionnaire©, a new tool to measure instrumental activities of daily living in dementia. Neuroepidemiology. 2013;41:35‐41. doi:10.1159/000346277 23712106

[53] Jutten RJ , Harrison JE , Brunner AJ , et al. The Cognitive‐Functional Composite is sensitive to clinical progression in early dementia: longitudinal findings from the Catch‐Cog study cohort. Alzheimers Dement (NY). 2020;6:e12020. doi:10.1002/trc2.12020 PMC7164406 32313832

[54] Jutten RJ , Dicks E , Vermaat L , et al. Impairment in complex activities of daily living is related to neurodegeneration in Alzheimer's disease–specific regions. Neurobiol Aging. 2019;75:109‐116. doi:10.1016/j.neurobiolaging.2018.11.018 30557769

[55] Verrijp M , Dubbelman MA , Visser LNC , et al. Everyday functioning in a community‐based volunteer population: differences between participant‐ and study partner‐report. Front Aging Neurosci. 2022;13. 10.3389/fnagi.2021.761932 PMC8767803 35069172

[56] Dubbelman MA , Verrijp M , Facal D , et al. The influence of diversity on the measurement of functional impairment: an international validation of the Amsterdam IADL Questionnaire in eight countries. Alzheimers Dement (Amst). 2020;12:e12021. doi:10.1002/dad2.12021 32420446 PMC7219786

[57] Dubbelman MA , Verrijp M , Terwee CB , et al. Determining the minimal important change of everyday functioning in dementia: pursuing clinical meaningfulness. Neurology. 2022;99:e954‐e964. doi:10.1212/WNL.0000000000200781 35641309 PMC9502738

[58] Kaufer DI , Cummings JL , Ketchel P , et al. Validation of the NPI‐Q, a brief clinical form of the Neuropsychiatric Inventory. J Neuropsychiatry Clin Neurosci. 2000;12:233‐239. doi:10.1176/jnp.12.2.233 11001602

[59] Cummings J . The Neuropsychiatric Inventory Questionnaire: background and administration 1994. (accessed July 23, 2020). https://www.alz.org/careplanning/downloads/npiq‐questionnaire.pdf

[60] Logsdon RG , Gibbons LE , McCurry SM , Teri L . Quality of life in Alzheimer's disease: patient and caregiver reports. J Ment Health Aging. 1999;5:21‐32.

[61] Logsdon RG , Gibbons LE , McCurry SM , Teri L . Assessing quality of life in older adults with cognitive impairment. Psychosom Med. 2002;64:510‐519. doi:10.1097/00006842-200205000-00016 12021425

[62] Ware J , Kosinski M , Keller SD . A 12‐Item Short‐Form Health Survey: construction of scales and preliminary tests of reliability and validity. Med Care. 1996;34:220‐233. doi:10.1097/00005650-199603000-00003 8628042

[63] Huo T , Guo Y , Shenkman E , Muller K . Assessing the reliability of the Short Form 12 (SF‐12) health survey in adults with mental health conditions: a report from the Wellness Incentive and Navigation (WIN) study. Health Qual Life Outcomes. 2018;16:34. doi:10.1186/s12955-018-0858-2 29439718 PMC5811954

[64] Rampakakis E , Ste‐Marie PA , Sampalis JS , Karellis A , Shir Y , Fitzcharles M‐A . Real‐life assessment of the validity of Patient Global Impression of Change in fibromyalgia. RMD Open. 2015;1:e000146. doi:10.1136/rmdopen-2015-000146 26535150 PMC4623367

[65] Viktrup L , Hayes RP , Wang P , Shen W . Construct validation of Patient Global Impression of Severity (PGI‐S) and Improvement (PGI‐I) questionnaires in the treatment of men with lower urinary tract symptoms secondary to benign prostatic hyperplasia. BMC Urol. 2012;12:30. doi:10.1186/1471-2490-12-30 23134716 PMC3503561

[66] Seng BK , Luo N , Ng WY , et al. Validity and reliability of the Zarit Burden Interview in assessing caregiving burden. Ann Acad Med Singap. 2010;39:758‐763. 21063635

[67] Wimo A , Jonsson L , Zbrozek A . The Resource Utilization in Dementia (RUD) instrument is valid for assessing informal care time in community‐living patients with dementia. J Nutr Health Aging. 2010;14:685‐690. doi:10.1007/s12603-010-0316-2 20922346

[68] Tsoy E , Zygouris S , Possin KL . Current state of self‐administered brief computerized cognitive assessments for detection of cognitive disorders in older adults: a systematic review. J Prev Alzheimers Dis. 2021;8:267‐276. doi:10.14283/jpad.2021.11 34101783 PMC7987552

