# Supplementary material for: Generating real‐world evidence in Alzheimer's disease: Considerations for establishing a core dataset
Source: Alzheimers Dement. 2024 May 6;20(6):4331–41. doi: 10.1002/alz.13785 (PMC11180865; doi:10.1002/alz.13785)
Supplement: Supplementary file 1 — Supporting Information [file ALZ-20-4331-s003.docx]

# SUPPLEMENTARY DATA S1: Administration, validity, and limitations of the proposed assessment tools

### S1.1 Montreal Cognitive Assessment (MoCA)

The MoCA is a screening tool completed by a trained clinician following an interview with the patient that takes around 10 minutes [1,2]. The 30-point assessment tests eight cognitive domains [3]. A score of ≥26 indicates normal cognitive function, and a score of ≤25 has been proposed to demonstrate cognitive impairment (18–25 may show mild cognitive impairment [MCI], 10–17 may show moderate cognitive impairment, and <10 may show severe cognitive impairment) [1,4]. However, if a patient scores in the 24–26 range (borderline performance), or the clinician requires confirmation of cognitive impairment, a more comprehensive neurocognitive or formal neuropsychological assessment should be considered [5].

The MoCA, which has excellent sensitivity for diagnosing MCI and Alzheimer’s disease (AD) dementia (90% and 100%, respectively), can be used when a patient first presents with cognitive deficit symptoms [4]. The MoCA is superior to the Mini-Mental State Examination (MMSE) at identifying MCI, with respective sensitivities of 90% and 18% [4,6]. It also has high test-retest reliability and internal consistency [4].

The MoCA is not as extensively studied as the MMSE [2], and a trained clinician is required for test administration and interpretation of results [1]. The assessment may take ≥10 minutes for individuals with severe impairment [2].

### S1.2 Quick Dementia Rating System (QDRS)

The QDRS is a rapid screening tool intended to capture the earliest signs of cognitive impairment [7]. The QDRS is completed by an informant/patient and can be administered in person or remotely within 3–5 minutes without requiring a trained clinician or participation of the patient [2,7,8]. To categorize changes in the patient’s cognitive and functional abilities, the QDRS assesses ten items comprising memory and recall; orientation; decision‑making and problem‑solving abilities; activities outside the home; function at home and hobbies; toileting and personal hygiene; behavior and personality changes; language and communication abilities; attention and concentration; and mood [2,9]. The total score is on a continuous scale of 0–30, with differentiation based on the following cutoff points: normal (0–1), MCI (2–5), mild dementia (6–12), moderate dementia (13–20), and severe dementia (20–30) [2]. Scores of ≥2 indicate impairment and that the individual should be further assessed to establish a formal diagnosis [9].

The QDRS can reliably discriminate between individuals with and without dementia [8] and accurately stage dementia [2]. It has excellent internal consistency and a strong correlation with the Clinical Dementia Rating Global Score and Clinical Dementia Rating Scale-Sum of Boxes [9]. The validity of the QDRS has been demonstrated in research and community real-world settings and demonstrates a good correlation with AD biomarkers; however, further validation in non-White populations is needed [8,10].

Other limitations of the QDRS include that the informant version is dependent on an observant informant [2], and the patient version has the potential for recall bias and minimization of deficits [9].

### S1.3 Amsterdam Instrumental Activities of Daily Living Questionnaire Short Version (A‑IADL‑Q‑SV)

Activities of Daily Living (ADLs) can be described as basic or instrumental (BADL/IADL); BADLs involve caring for oneself, such as personal care, mobility, and eating. IADLs are more complex daily activities requiring greater cognitive organization than BADLs and tend to be affected in early-stage AD, typically before any impairment in BADLs is noted [11].

The original Amsterdam Instrumental Activities of Daily Living Questionnaire (A-IADL-Q) consists of 70 items in eight categories (household, administration, work, computer use, leisure time, appliances, transport, and other activities) and was developed to evaluate IADLs and assess difficulties with complex daily activities [12,13]. A short version, the A‑IADL‑Q-SV, takes 10–15 minutes to complete, and it consists of 30 items in the same eight categories as the A-IADL-Q. The test is completed by the patient’s caregiver, and each item is rated for difficulty on a 5‑point scale ranging from ‘no difficulty’ to ‘unable to perform’, with scoring based on the Item Response Theory [12].

Despite being significantly shorter, the A‑IADL‑Q-SV maintains the psychometric qualities of the original version. The A‑IADL‑Q-SV has adequate measurement precision along the entire spectrum of IADL functioning and high internal consistency. It also has a high concordance with the original A-IADL-Q and the MMSE, which supports the validity of the construct [12].

Proxy-based IADL measures may, however, be confounded by respondent influences, such as caregiver burden or depression, and are dependent on an observant informant [12,13]. Furthermore, as the A‑IADL‑Q-SV was developed and validated as a digital assessment, a suitable software/platform is required [13]. Lastly, further research is needed to examine if the A‑IADL‑Q-SV is sensitive to changes over time within patients [12].

### S1.4 Neuropsychiatric Inventory Questionnaire (NPI‑Q)

The Neuropsychiatric Inventory (NPI), an informant‑based interview, was developed to rapidly assess a wide range of behaviors encountered in patients with dementia [14]. The NPI‑Q is a simplified, self‑administered version of the NPI and takes less than 5 minutes to administer [15]. It is used as a brief screening tool to identify changes in behavior in patients with memory disorders and their associated impact on caregivers [16]. The NPI-Q consists of 12 questions assessing multiple neuropsychiatric domains, including delusions, hallucinations, agitation/aggression, depression/dysphoria, anxiety, elation/euphoria, apathy/indifference, disinhibition, irritability/liability, motor disturbance, night‑time behaviors, and appetite/eating [15], which are more common in MCI and AD dementia than in cognitively normal older adults [17–19]. The total severity score ranges from 0 to 36, and the total caregiver distress score ranges from 0 to 60; higher scores indicate greater neuropsychiatric symptom severity and higher caregiver distress and can determine the need for directed interventions involving the patient and/or caregiver [16].

The NPI-Q has adequate test-retest reliability and convergent validity with respect to symptom domain scores and caregiver distress ratings on the NPI [16].

As with all informant-based assessments, the NPI-Q is dependent on an observant informant [15]. Further research is needed on the NPI-Q in clinical practice settings involving more ethnically and demographically diverse populations. Although the format and wording of the assessment were designed to be simple, some informants may have difficulty in completing the self-administered form, and as such, an abbreviated interview may be required [16].

### S1.5 Quality of Life in Alzheimer’s Disease (QOL-AD)

The QOL-AD is a 13-item questionnaire designed to measure various domains of quality of life (QOL) (e.g., physical health, mood, relationships, activities, and ability to complete tasks) and can be completed in approximately 10 minutes, either by patient self‑reporting or proxy-reporting if the patient has more advanced stages of dementia. The questionnaire is answered using a 4-point scale (poor, fair, good, or excellent), with total scores ranging from 13 to 52; higher scores indicate greater QOL [20,21].

The QOL-AD has acceptable internal consistency, high test-retest reliability, and good construct validity, especially with regard to divergent validity [20].

However, the QOL-AD relies on a conceptualization of QOL that could be seen by some as broad, and there is evidence that QOL ratings differ between patients and caregivers [20].

### S1.6 12-item Short Form Survey (SF-12)

Originally derived from the Medical Outcomes Study (MOS) 36-item Short Form Survey (SF‑36), the SF-12 is a structured, self-reported questionnaire with 12 questions covering eight health domains, which takes around 2 minutes to complete [22,23]. It is scored using the MOS software program that creates two summary scores (physical and mental health) using the weighted means of the eight domains [23,24].

The SF-12 has been demonstrated to have good reliability and a high degree of correlation with SF-36 [22,23].

### S1.7 Patient Global Impression of Severity (PGI-S) and Patient Global Impression of Change (PGI-C)

The PGI-S and PGI-C are both 1-item questionnaires designed to assess patients’ overall perception of a specific condition and have been used in various diseases [25,26]. The PGI-S uses a 4-point Likert scale to ask the patient to rate the severity of their AD, and the PGI-C uses a 7-point Likert scale to ask the patient to rate change in their AD [25–27]; this scale aims to quantify disease activity relative to an anchor point (specifically, the difference between their current and previous health state) [25].

These assessments have moderate-to-high correlation with other patient-reported measures, including across variations in ethnicity [26].

The PGI-S and PGI-C only provide an overall appraisal of a patient’s condition; multi-item instruments may be required to fully describe the impact of treatment on various symptoms [26]

### S1.8 Zarit Burden Interview (ZBI)

The 22-item ZBI is a self-report questionnaire for measuring the caregiver’s perceived burden of providing care, with questions focusing on health, psychological well-being, finances, social life, and relationships [28]. It takes approximately 15 minutes to complete and is rated on a 5‑point Likert scale ranging from 0 (never) to 4 (nearly always); the sum of scores ranges between 0 and 88, with higher scores indicating greater burden [28,29].

The ZBI is validated in many culturally and ethnically diverse populations and has a high correlation with other standardized instruments. It has adequate test-retest reliability and internal consistency [28].

A limitation of the ZBI is positive aspects of caregiving that might reduce feelings of burden are not explored. Further research is required to validate this questionnaire in non-family caregivers [28].

### S1.9 Resource Utilization in Dementia (RUD) Lite

The RUD instrument was developed to assess the use of resources among patients with dementia in clinical trial settings, enabling the comparison of costs and care across many different countries [30,31]. The RUD Lite questionnaire is a shorter version of the RUD, taking around 15 minutes to complete, and involves 25 items encompassing healthcare resource utilization and caregiver time spent on formal and informal care [32]. Research has shown the RUD Lite is a valid and accurate measure of caregiver time [30].

# SUPPLEMENTARY DATA S1: References

[1] Nasreddine ZS. MoCA test FAQ 2019. https://www.mocatest.org/faq/ (accessed October 9, 2020).

[2] Galvin JE. Using informant and performance screening methods to detect mild cognitive impairment and dementia. Curr Geriatr Rep 2018;7:19–25. https://doi.org/10.1007/s13670-018-0236-2.

[3] MoCA Cognition. The MoCA test 2023. https://www.mocatest.org/the-moca-test/ (accessed March 26, 2020).

[4] Nasreddine ZS, Phillips NA, Bédirian V, Charbonneau S, Whitehead V, Collin I, et al. The Montreal Cognitive Assessment, MoCA: a brief screening tool for mild cognitive impairment. J Am Geriatr Soc 2005;53:695–9. https://doi.org/10.1111/j.1532-5415.2005.53221.x.

[5] Thomann AE, Berres M, Goettel N, Steiner LA, Monsch AU. Enhanced diagnostic accuracy for neurocognitive disorders: a revised cut-off approach for the Montreal Cognitive Assessment. Alzheimers Res Ther 2020;12:39. https://doi.org/10.1186/s13195-020-00603-8.

[6] Pinto TC, Machado L, Bulgacov TM, Rodrigues-Júnior AL, Costa ML, Ximenes RC, et al. Is the Montreal Cognitive Assessment (MoCA) screening superior to the Mini-Mental State Examination (MMSE) in the detection of mild cognitive impairment (MCI) and Alzheimer’s Disease (AD) in the elderly? Int Psychogeriatr 2019;31:491–504. https://doi.org/10.1017/S1041610218001370.

[7] University of Miami Comprehensive Center for Brain Health. The Quick Dementia Rating System (QDRS) – patient and informant versions 2023. https://umiamibrainhealth.org/downloads/the-quick-dementia-rating-system-qdrs-patient-and-informant-versions/ (accessed March 19, 2023).

[8] Galvin JE. The quick dementia rating system (QDRS): a rapid dementia staging tool. Alzheimers Dement Amst 2015;1:249–59. https://doi.org/10.1016/j.dadm.2015.03.003.

[9] Galvin JE, Tolea MI, Chrisphonte S. Using a patient-reported outcome to improve detection of cognitive impairment and dementia: the patient version of the Quick Dementia Rating System (QDRS). PloS One 2020;15:e0240422. https://doi.org/10.1371/journal.pone.0240422.

[10] Duff K, Wan L, Levine DA, Giordani B, Fowler NR, Fagerlin A, et al. The quick dementia rating system and its relationship to biomarkers of Alzheimer’s disease and neuropsychological performance. Dement Geriatr Cogn Disord 2022;51:214–20. https://doi.org/10.1159/000524548.

[11] Mlinac ME, Feng MC. Assessment of activities of daily living, self-care, and independence. Arch Clin Neuropsychol 2016;31:506–16. https://doi.org/10.1093/arclin/acw049.

[12] Jutten RJ, Peeters CF, Leijdesdorff SM, Visser PJ, Maier AB, Terwee CB, et al. Detecting functional decline from normal aging to dementia: development and validation of a short version of the Amsterdam IADL Questionnaire. Alzheimers Dement Amst 2017;8:26–35. https://doi.org/10.1016/j.dadm.2017.03.002.

[13] Sikkes SAM, de Lange-de Klerk ESM, Pijnenburg YAL, Gillissen F, Romkes R, Knol DL, et al. A new informant-based questionnaire for instrumental activities of daily living in dementia. Alzheimers Dement 2012;8:536–43. https://doi.org/10.1016/j.jalz.2011.08.006.

[14] Cummings JL, Mega M, Gray K, Rosenberg-Thompson S, Carusi DA, Gornbein J. The Neuropsychiatric Inventory: comprehensive assessment of psychopathology in dementia. Neurology 1994;44:2308–14. https://doi.org/10.1212/wnl.44.12.2308.

[15] Cummings J. The Neuropsychiatric Inventory Questionnaire: background and administration 1994. https://www.alz.org/careplanning/downloads/npiq-questionnaire.pdf (accessed July 23, 2020).

[16] Kaufer DI, Cummings JL, Ketchel P, Smith V, MacMillan A, Shelley T, et al. Validation of the NPI-Q, a brief clinical form of the Neuropsychiatric Inventory. J Neuropsychiatry Clin Neurosci 2000;12:233–9. https://doi.org/10.1176/jnp.12.2.233.

[17] Geda YE, Roberts RO, Knopman DS, Petersen RC, Christianson TJH, Pankratz VS, et al. Prevalence of neuropsychiatric symptoms in mild cognitive impairment and normal cognitive aging: population-based study. Arch Gen Psychiatry 2008;65:1193–8. https://doi.org/10.1001/archpsyc.65.10.1193.

[18] Steinberg M, Shao H, Zandi P, Lyketsos CG, Welsh-Bohmer KA, Norton MC, et al. Point and 5-year period prevalence of neuropsychiatric symptoms in dementia: the Cache County Study. Int J Geriatr Psychiatry 2008;23:170–7. https://doi.org/10.1002/gps.1858.

[19] Yang A-N, Wang X-L, Rui H-R, Luo H, Pang M, Dou X-M. Neuropsychiatric symptoms and risk factors in mild cognitive impairment: a cohort investigation of elderly patients. J Nutr Health Aging 2020;24:237–41. https://doi.org/10.1007/s12603-020-1312-9.

[20] Stypa V, Haussermann P, Fleiner T, Neumann S. Validity and reliability of the German Quality of Life-Alzheimer’s Disease (QoL-AD) self-report scale. J Alzheimers Dis 2020;77:581–90. https://doi.org/10.3233/JAD-200400.

[21] Kahle-Wrobleski K, Ye W, Henley D, Hake AM, Siemers E, Chen Y-F, et al. Assessing quality of life in Alzheimer’s disease: implications for clinical trials. Alzheimers Dement Amst 2016;6:82–90. https://doi.org/10.1016/j.dadm.2016.11.004.

[22] Ware J, Kosinski M, Keller SD. A 12-Item Short-Form Health Survey: construction of scales and preliminary tests of reliability and validity. Med Care 1996;34:220–33. https://doi.org/10.1097/00005650-199603000-00003.

[23] Huo T, Guo Y, Shenkman E, Muller K. Assessing the reliability of the short form 12 (SF-12) health survey in adults with mental health conditions: a report from the wellness incentive and navigation (WIN) study. Health Qual Life Outcomes 2018;16:34. https://doi.org/10.1186/s12955-018-0858-2.

[24] Larson CO. Use of the SF-12 instrument for measuring the health of homeless persons. Health Serv Res 2002;37:733–50. https://doi.org/10.1111/1475-6773.00046.

[25] Rampakakis E, Ste-Marie PA, Sampalis JS, Karellis A, Shir Y, Fitzcharles M-A. Real-life assessment of the validity of patient global impression of change in fibromyalgia. RMD Open 2015;1:e000146. https://doi.org/10.1136/rmdopen-2015-000146.

[26] Viktrup L, Hayes RP, Wang P, Shen W. Construct validation of patient global impression of severity (PGI-S) and improvement (PGI-I) questionnaires in the treatment of men with lower urinary tract symptoms secondary to benign prostatic hyperplasia. BMC Urol 2012;12:30. https://doi.org/10.1186/1471-2490-12-30.

[27] Ferguson L, Scheman J. Patient global impression of change scores within the context of a chronic pain rehabilitation program. J Pain 2009;10(4 Suppl.):S73 (Abstract 397). https://doi.org/10.1016/j.jpain.2009.01.258.

[28] Seng BK, Luo N, Ng WY, Lim J, Chionh HL, Goh J, et al. Validity and reliability of the Zarit Burden Interview in assessing caregiving burden. Ann Acad Med Singap 2010;39:758–63.

[29] Cifu DX, Carne W, Brown R, Pegg P, Ong J, Qutubuddin A, et al. Caregiver distress in parkinsonism. J Rehabil Res Dev 2006;43:499–508. https://doi.org/10.1682/JRRD.2005.08.1365.

[30] Wimo A, Jonsson L, Zbrozek A. The Resource Utilization in Dementia (RUD) instrument is valid for assessing informal care time in community-living patients with dementia. J Nutr Health Aging 2010;14:685–90. https://doi.org/10.1007/s12603-010-0316-2.

[31] Wimo A, Gustavsson A, Jönsson L, Winblad B, Hsu M-A, Gannon B. Application of Resource Utilization in Dementia (RUD) instrument in a global setting. Alzheimers Dement 2013;9:429–35. https://doi.org/10.1016/j.jalz.2012.06.008.

[32] Casey D, Gallagher N, Devane D, Woods B, Murphy K, Smyth S, et al. The feasibility of a Comprehensive Resilience-building psychosocial Intervention (CREST) for people with dementia in the community: protocol for a non-randomised feasibility study. Pilot Feasibility Stud 2020;6:177. https://doi.org/10.1186/s40814-020-00701-2.

## SUPPLEMENTARY DATA S1: Abbreviations

A-IADL-Q, Amsterdam Instrumental Activities of Daily Living Questionnaire

A-IADL-Q-SV, Amsterdam Instrumental Activities of Daily Living Questionnaire Short Version

AD, Alzheimer’s disease

ADL, Activities of Daily Living

BADL, basic ADL

IADL, Instrumental ADL

MCI, mild cognitive impairment

MMSE, Mini-Mental State Examination

MoCA, Montreal Cognitive Assessment

MOS, Medical Outcomes Study

NPI, Neuropsychiatric Inventory

NPI-Q, Neuropsychiatric Inventory Questionnaire

PGI-C, Patient Global Impression of Change

PGI-S, Patient Global Impression of Severity

QDRS, Quick Dementia Rating System

QOL, quality of life

QOL-AD, Quality of Life in Alzheimer’s Disease

RUD, Resource Utilization in Dementia

SF-12, 12-item Short Form Survey

SF-36, 36-item Short Form Survey

ZBI, Zarit Burden Interview
