# Supplementary material for: Generating real‐world evidence in Alzheimer's disease: Considerations for establishing a core dataset
Source: Alzheimers Dement. 2024 May 6;20(6):4331–41. doi: 10.1002/alz.13785 (PMC11180865; doi:10.1002/alz.13785)
Supplement: Supplementary file 2 — Table S1 [file ALZ-20-4331-s001.docx]

## SUPPLEMENTARY TABLE 1: Detailed schedule of assessments – baseline and longitudinal datasets

| **Assessment** | **Baseline dataset** | **Longitudinal dataset** |
| --- | --- | --- |
| **Demographic data** | | |
| ICF | X |  |
| Specialty of physician prescribing drug | X |  |
| AD care setting | O |  |
| Setting of drug administration | O |  |
| Birth year | X |  |
| Sex | X |  |
| Primary language | X |  |
| Race | X |  |
| Ethnicity | X |  |
| Place of residence | X | X |
| Level of care | X | X |
| Years of formal education | O |  |
| Marital status | O |  |
| Family history of AD | X |  |
| Living situation | X | X |
| Insurance status | X | X |
| **AD clinical characteristics** | | |
| Age at / date of onset | X |  |
| Age at / date of diagnosis | X |  |
| Clinical disease stage* | X | X |
| *APOE* ε4 genotyping^†^ | O^‡^ | O^‡^ |
| **Medical history** | | |
| Diabetes | X | O^‡^ |
| Bleeding disorders | X | O^‡^ |
| Hypercoagulable states | X | O^‡^ |
| Cancer | X | O^‡^ |
| Coronary artery disease | X | O^‡^ |
| Congestive heart failure | X | O^‡^ |
| Hypertension | X | O^‡^ |
| Cirrhosis | X | O^‡^ |
| Major depression | X | O^‡^ |
| Chronic headaches | X | O^‡^ |
| Stroke | X | X |
| Transient ischemic attack | X | X |
| History of seizure | X | X |
| Other neurological disease(s) | X | X |
| **AE** | | |
| NSAEs |  | X |
| SAEs |  | X |
| ARIA-related clinical AEs per investigator |  | X |
| AEs leading to discontinuation of drug |  | X |
| Other neurological disease(s) or AEs | X | X |
| **Biomarker data for Aβ confirmation** | | |
| Aβ measurement (CSF or PET) | X |  |
| CSF biomarker results (if Aβ measurement is CSF analysis) | X |  |
| **Biomarker data for future analysis** | | |
| Blood (plasma and serum) collection for biobank | X | X |
| CSF collection or sharing of CSF biomarker data | O | O |
| Donation of blood for future genetic testing | O | O |
| **Concomitant medications** | | |
| Medications prescribed | X | O^‡^ |
| Medication count | X | X |
| Change in medication |  | X |
| **Treatment dosing** | | |
| Date of initiation | X |  |
| Treatment details (dosing) | X | X |
| Status of treatment | X | X |
| Reason for discontinuation, if applicable | O | O |
| Titration visit |  | X |
| **Brain imaging data** | | |
| MRI brain | X | X |
| ARIA-E | X | X |
| ARIA-H micro-hemorrhages  (≤1 cm in diameter) count | X | X |
| ARIA-H superficial siderosis | X | X |
| Hemorrhages >1 cm in diameter | X | X |
| White matter T2 hyperintense lesions | O | O |
| Lacunar infarct (≤1.5 cm in diameter) | O | O |
| Ischemic infarct (>1.5 cm in diameter, irrespective of anatomical location) | O | O |
| **Physical assessments** | | |
| BMI / height and weight | X | X |
| Vital signs | X | X |
| Neurological examination | O | O |
| **Lifestyle data** | | |
| Alcohol use | X | O^‡^ |
| Cigarette smoking | X | O^‡^ |
| Substance abuse | X | O^‡^ |
| Physical exercise | O^‡^ | O^‡^ |
| **Cognitive assessments** | | |
| CDR (for AD staging) | X |  |
| MoCA (version 8.1) | X | X |
| QDRS‑IV/PV | X | X |
| **Functional and neuropsychiatric assessments** | | |
| FAQ | X | X |
| A-IADL-Q-SV | X | X |
| GDS-SF | X | X |
| NPI-Q | X | X |
| **Quality of life and disease burden assessments** | | |
| 13-item QOL-AD | X | X |
| SF-12 (version 2) | X | X |
| ZBI (22 items) | X | X |
| RUD Lite | X | X |
| PGI-S | X |  |
| PGI-C |  | X |

*Disease stage to be determined based on clinical assessment by the investigator/treating physician or neurologist; ^†^the homozygous/heterozygous *APOE* ε4 genotype is associated with an increased risk of ARIA associated with second-generation anti-Aβ monoclonal antibodies [1]; ^‡^can be considered mandatory if it does not make the study too burdensome.

X denotes core data elements; O denotes optional data elements.

Abbreviations: Aβ, amyloid beta; A‑IADL‑Q‑SV, Amsterdam Instrumental Activities of Daily Living Questionnaire Short Version; AD, Alzheimer’s disease; AE, adverse event; *APOE* ε4, apolipoprotein E ε4; ARIA, amyloid-related imaging abnormalities; ARIA‑E, amyloid-related imaging abnormalities due to vasogenic edema; ARIA-H, amyloid-related imaging abnormalities due to micro-hemorrhages, macro-hemorrhages; BMI, body mass index; CDR, Clinical Dementia Rating; CSF, cerebrospinal fluid; FAQ, Functional Activities Questionnaire; GDS‑SF, Geriatric Depression Scale Short Form; ICF, informed consent form; MoCA, Montreal Cognitive Assessment; MRI, magnetic resonance imaging; NPI-Q, Neuropsychiatric Inventory Questionnaire; NSAE, non-serious adverse event; PET, positron emission tomography; PGI-C, Patient Global Impression of Change; PGI-S, Patient Global Impression of Severity; QDRS‑IV/PV, Quick Dementia Rating System Informant Version / Patient Version; QOL-AD, Quality of Life in Alzheimer’s Disease; RUD, Resource Utilization in Dementia; SAE, serious adverse event; SF‑12, 12-item Short Form Survey; ZBI, Zarit Burden Interview.

## SUPPLEMENTARY TABLE 1: References

[1] Withington CG, Turner RS. Amyloid-related imaging abnormalities with anti-amyloid antibodies for the treatment of dementia due to Alzheimer's disease. Front Neurol 2022;13:862369. https://doi.org/10.3389/fneur.2022.862369.
